# External validation of urinary C–C motif chemokine ligand 14 (CCL14) for prediction of persistent acute kidney injury

**DOI:** 10.1186/s13054-021-03618-1

**Published:** 2021-05-31

**Authors:** Sean M. Bagshaw, Ali Al-Khafaji, Antonio Artigas, Danielle Davison, Michael Haase, Matthew Lissauer, Kai Zacharowski, Lakhmir S. Chawla, Thomas Kwan, J. Patrick Kampf, Paul McPherson, John A. Kellum

**Affiliations:** 1 Department of Critical Care Medicine, Faculty of Medicine and Dentistry, University of Alberta, 2-124 Clinical Sciences Building, 8440-112 ST NW, Edmonton, AB T6G 2B7 Canada; 2 Department of Critical Care Medicine, Center for Critical Care Nephrology, University of Pittsburgh, 3550 Terrace St., Scaife Hall, Suite 600, Pittsburgh, PA 15213 USA; 3 Critical Care Department, Corporacion Sanitaria Universitaria Parc Tauli, CIBER Enfermedades Respiratorias, Autonomous University of Barcelona, Parc Tauli 1, 08208 Sabadell, Spain; 4 Department of Anesthesiology and Critical Care Medicine, School of Medicine and Health Sciences, George Washington University, 900 23rd St. NW, Washington, DC 20037 USA; 5 Diaverum Renal Care Center, 14469 Potsdam, Germany and Medical Faculty, Otto Von-Guericke-University Magdeburg, Leipziger Str. 44, 39120 Magdeburg, Germany; 6 Division of Acute Care Surgery, Department of Surgery, Rutgers-Robert Wood Johnson Medical School, 125 Patterson Street, New Brunswick, NJ 07746 USA; 7 Department of Anesthesiology, Intensive Care Medicine and Pain Therapy, University Hospital Frankfurt, Goethe University, Theodor-Stern-Kai 7, 60590 Frankfurt am Main, Germany; 8 Veterans Affairs Medical Center, 3350 La Jolla Village Dr, San Diego, CA 92161 USA; 9 Astute Medical, Inc. (a bioMérieux company), 3550 General Atomics Ct, San Diego, CA 92121 USA

**Keywords:** Acute kidney injury, Prediction, Validation, Renal replacement therapy, Mortality

## Abstract

**Background:**

Persistent acute kidney injury (AKI) portends worse clinical outcomes and remains a therapeutic challenge for clinicians. A recent study found that urinary C–C motif chemokine ligand 14 (CCL14) can predict the development of persistent AKI. We aimed to externally validate urinary CCL14 for the prediction of persistent AKI in critically ill patients.

**Methods:**

This was a secondary analysis of the prospective multi-center SAPPHIRE study. We evaluated critically ill patients with cardiac and/or respiratory dysfunction who developed Kidney Disease: Improving Global Outcomes (KDIGO) stage 2–3 AKI within one week of enrollment. The main exposure was the urinary concentration of CCL14 measured at the onset of AKI stage 2–3. The primary endpoint was the development of persistent severe AKI, defined as  ≥ 72 h of KDIGO stage 3 AKI or death or renal-replacement therapy (RRT) prior to 72 h. The secondary endpoint was a composite of RRT and/or death by 90 days. We used receiver operating characteristic (ROC) curve analysis to assess discriminative ability of urinary CCL14 for the development of persistent severe AKI and multivariate analysis to compare tertiles of urinary CCL14 and outcomes.

**Results:**

We included 195 patients who developed KDIGO stage 2–3 AKI. Of these, 28 (14%) developed persistent severe AKI, of whom 15 had AKI  ≥ 72 h, 12 received RRT and 1 died prior to  ≥ 72 h of KDIGO stage 3 AKI. Persistent severe AKI was associated with chronic kidney disease, diabetes mellitus, higher non-renal APACHE III score, greater fluid balance, vasopressor use, and greater change in baseline serum creatinine. The AUC for urinary CCL14 to predict persistent severe AKI was 0.81 (95% CI, 0.72–0.89). The risk of persistent severe AKI increased with higher values of urinary CCL14. RRT and/or death at 90 days increased within tertiles of urinary CCL14 concentration.

**Conclusions:**

This secondary analysis externally validates urinary CCL14 to predict persistent severe AKI in critically ill patients.

**Supplementary Information:**

The online version contains supplementary material available at 10.1186/s13054-021-03618-1.

## Background

Acute kidney injury (AKI) is a common and vexing clinical challenge [1]. For most patients, kidney dysfunction with AKI either resolves rapidly or improves along with resolution of their acute illness [2]; however, in up to one-third of patients, AKI fails to resolve [3]. This persistent AKI has prognostic importance and portends high risk of long-term sequelae, including incident chronic kidney disease (CKD) and reduced survival [2]. Unfortunately, there are no interventions proven to modify the clinical course for patients with persistent AKI [4]. This is likely attributable to the inability to predict those at high risk for development of persistent AKI, a factor that has undoubtedly hampered suitable patient selection for clinical trials and contributed to therapeutic uncertainty for clinicians [5].

Recently, the RUBY study described the discovery of urinary C–C motif chemokine ligand-14 (CCL14) to predict the development of persistent severe AKI, defined as Kidney Disease: Improving Global Outcomes (KDIGO) stage 3 AKI for 72 h or greater, among 331 critically ill patients with KDIGO stage 2 or greater AKI (AUC 0.83) [6]. In this study, urinary CCL14 was found to outperform several other AKI biomarkers to predict persistent severe AKI. CCL14 is a member of the chemokine family recognized for its role in macrophage trafficking and may contribute to the development of persistent kidney damage, maladaptive repair, and risk for non-recovery of kidney function [6, 7].

Knowledge of the risk for patients to develop persistent AKI would have important implications for bedside care, including decision making on the utilization of medications with potentially direct toxic or accumulative effects and the informed application of renal-replacement therapy (RRT) [8]. Moreover, such knowledge would provide insights into the pathobiology and kinetics of kidney repair and recovery and further enable predictive enrichment in randomized trials of potential therapeutic targets [5]. Accordingly, we aimed to externally validate the performance of the novel biomarker urinary CCL14 for the prediction of persistent severe AKI among an established, heterogenous cohort of critically ill patients.

## Methods

The SAPPHIRE study protocol was approved by the Western Institutional Review Board (Olympia, Washington, USA) and by the investigational review boards/ethics committees as required by each participating institution [9]. All enrolled patients or their legally authorized representatives provided written informed consent.

### Design

This was a secondary analysis of the previously described SAPPHIRE study, a multi-national prospective observational cohort study of heterogeneous critically ill patients at risk for development of AKI [9].

### Patients

Patients enrolled in the SAPPHIRE study served as the source population for the current study [9]. SAPPHIRE enrolled adult critically ill patients with cardiac or respiratory dysfunction without known stage 2–3 AKI at the time of enrollment. The subset of SAPPHIRE patients who developed stage 2–3 AKI within one week of enrollment were included in the present analysis. Aligned with the eligibility in the RUBY study, no patients in this SAPPHIRE cohort had received a kidney transplant, had prior receipt of RRT or had active HIV or hepatitis infection [6]. Serial serum creatinine and urine output data, mortality, and RRT utilization were abstracted from the medical record for ascertainment of AKI stage, using the full KDIGO classification criteria (serum creatinine and urine output) and for endpoint determination. The reference (baseline) serum creatinine was obtained by priority in the following sequence: (1) if at least five values were available, the median of all values available from six months to six days prior to enrollment was used; (2) if not available, the lowest value in the five days prior to enrollment was used; and (3) if no pre-enrollment creatinine was available, the creatinine value at the time of enrollment was used [9].

### Main exposure

The main exposure was the urinary concentration of CCL14, measured within 36 h of the onset of when stage 2 or greater AKI was first identified.

### Clinical endpoints

The primary endpoint was the development of persistent severe AKI, using both serum creatinine and urine output criteria, as previously defined in the RUBY study [6]. Briefly, patients who developed 72 consecutive hours of stage 3 AKI or who died or received RRT prior to achieving 72 consecutive hours of stage 3 AKI were considered endpoint positive. Patients with stage 2 AKI at the time of sample collection for biomarker testing were considered endpoint positive if persistent stage 3 AKI started within 48 h of sample collection. This aligns with recent randomized trials evaluating timing of RRT initiation that integrated persistent AKI at 72 h as indications for starting RRT [8]. The secondary endpoint was a composite of RRT and/or death by 90 days.

### Sample, data collection and measurement

We collected urine samples twice daily for 4 days from enrollment and then once daily for 3 days. Supernatants from centrifuged urine samples were flash frozen, stored at or below − 70 °C, and thawed prior to analysis. To align with the inclusion criteria for the RUBY study, all urine samples within 36 h of onset of the first instance of stage 2 AKI (or stage 3 AKI if not preceded by stage 2) and prior to initiation of RRT were analyzed. An average of 2.5 urine samples per patient were included in the analysis. Technicians blinded to the clinical data measured CCL14 concentrations in the urine samples by immunoassay using the NEPHROCLEAR™ CCL14 Test on the Astute140® Meter (Astute Medical, San Diego, CA).

### Statistical analysis

We used receiver operating characteristic (ROC) curve analysis to assess discriminative ability of urinary CCL14 for the development of persistent severe AKI. The empirical area under the ROC curve (AUC) was calculated, and corresponding confidence interval (CI) was determined using bootstrap sampling at the patient level to account for multiple samples analyzed per patient. The AUC from the SAPPHIRE analysis was compared to the AUC previously reported for the same endpoint in the RUBY study using a permutation test [6]. We assessed the improvement of risk prediction when adding urinary CCL14 to a reference model consisting of baseline serum creatinine and non-renal Acute Physiology and Chronic Health Evaluation (APACHE) III score by integrated discrimination improvement (IDI), category-free net reclassification improvement (cfNRI), and AUC difference. Risk estimates for both the new and reference models were calculated using Generalized Estimating Equations (GEE) implemented in the geepack R package [10]. To assess the relationship between risk of persistent severe AKI and urinary CCL14 concentration, we plotted risk as a function of the measured urinary CCL14 concentration. We defined risk as the proportion of patient samples that were positive for persistent severe AKI among all patient samples with a urinary CCL14 concentration above the measured value. Risk curves were simulated for different prevalence levels of persistent severe AKI based on the established relationship between positive predictive value and disease prevalence [11]. We calculated hazard ratios for the composite endpoint of RRT and/or death within 90 days, both unadjusted and adjusted for baseline serum creatinine and non-renal APACHE III score, using Cox proportional hazards analysis with CCL14 concentrations as a time-dependent covariate. Probability (1–survival) of the composite of RRT and/or death within 90 days was plotted by CCL14 concentration of the first urine sample collected after onset of stage 2–3 AKI stratified into tertiles. We compared curves across tertiles using the log-rank test. Continuous and categorical baseline variables were compared between patients negative and positive for the primary endpoint using the Mann–Whitney U test and Fisher exact test, respectively. Continuous predictors in GEE and Cox regressions were standardized by subtracting the group mean and normalizing by the standard deviation. For all analyses, two-sided *p* values < 0.05 were considered statistically significant. We performed all analyses using R 4.0.2 (R Foundation for Statistical Computing. Vienna, Austria).

## Results

**Fig. 1:**
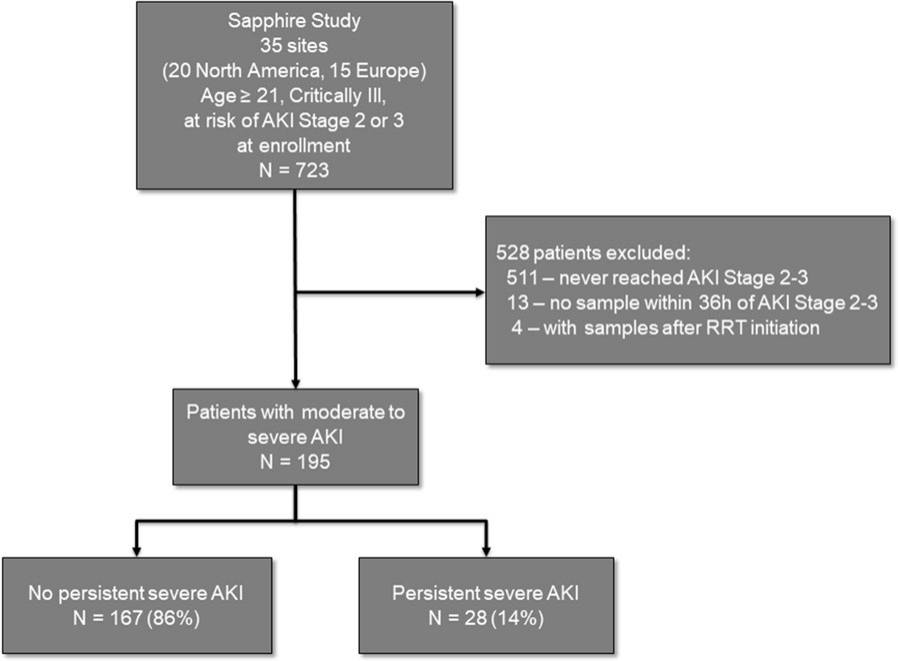
Patient cohort in SAPPHIRE study. Patients with at least one sample that met the persistent severe AKI criteria were considered endpoint positive

Of 723 patients enrolled in the SAPPHIRE study, 195 developed stage 2–3 AKI and were included in the analysis (Fig. 1). Of these, 28 (14%) developed the primary endpoint of persistent severe AKI, including 15 who had AKI ≥ 72 h, 12 who received RRT and 1 who died prior to achieving 72 consecutive hours in stage 3 AKI.

### Clinical characteristics

Patients who developed persistent severe AKI had greater prevalence of chronic kidney disease (CKD) and diabetes mellitus (DM) at baseline (Table 1). Persistent severe AKI was associated with higher non-renal APACHE III scores, greater positive fluid balance, and more vasopressor use at enrollment. Persistent severe AKI patients had higher baseline serum creatinine values, greater relative change and differences in serum creatinine compared with urine output and were more likely to have stage 3 AKI at enrollment compared to those with no persistent severe AKI (Additional file 1: S1 and S2).

**Table 1:** Baseline characteristics for all patients and those who met and did not meet the primary endpoint of persistent severe AKI

	All patients	No persistent severe AKI	Persistent severe AKI	*p* value^c^
Patients	195	167	28	
Male	113 (58%)	95 (57%)	18 (64%)	0.538
Age^a^ (years)	66 (56–75)	66 (56–75)	68.5 (58–76)	0.592
Race				
White or Caucasian	148 (76%)	126 (75%)	22 (79%)	
Black or African American	25 (13%)	23 (14%)	2 (7%)	
Other/unknown	22 (11%)	18 (11%)	4 (14%)	0.607
Chronic comorbidities				
Chronic kidney disease	21 (11%)	12 (7%)	9 (32%)	**< 0.001**
Diabetes mellitus	77 (39%)	59 (35%)	18 (64%)	**0.006**
Heart failure	43 (22%)	37 (22%)	6 (21%)	1.000
Coronary artery disease	70 (36%)	55 (33%)	15 (54%)	0.054
Hypertension	141 (72%)	118 (71%)	23 (82%)	0.258
COPD	47 (24%)	42 (25%)	5 (18%)	0.482
Cancer	54 (28%)	48 (29%)	6 (21%)	0.500
Reason for ICU admission				
Respiratory	90 (46%)	76 (46%)	14 (50%)	0.687
Surgery	67 (34%)	58 (35%)	9 (32%)	0.834
Cardiovascular	67 (34%)	54 (32%)	13 (46%)	0.196
Sepsis	42 (22%)	36 (22%)	6 (21%)	1.000
Neurological	18 (9%)	18 (11%)	0 (0%)	0.081
Trauma	7 (4%)	6 (4%)	1 (4%)	1.000
Other	30 (15%)	24 (14%)	6 (21%)	0.394
Baseline serum creatinine (mg/dL)	0.9 (0.7–1.2)	0.9 (0.7–1.2)	1.2 (0.8–2.2)	**0.006**
Maximum KDIGO stage before sample collection				
Stage 2	172 (88%)	160 (96%)	12 (43%)	
Stage 3	23 (12%)	7 (4%)	16 (57%)	**< 0.001**
eGFR < 60 mL/min	88 (45%)	68 (42%)	20 (74%)	**0.003**
Diuretics	75 (38%)	63 (38%)	12 (43%)	0.676
Vasopressor	101 (52%)	80 (48%)	21 (75%)	**0.008**
Mechanical ventilation	164 (84%)	138 (83%)	26 (93%)	0.263
Fluid balance (mL)^a^	2044 (248–3362)	1879 (180–3154)	3226 (695–4554)	**0.025**
Non-renal APACHE III score	62 (43–86)	60 (42–85)	80 (58–104)	**0.002**
Time from ICU admission to enrollment (h)	14 (6–20)	14 (6–20)	14 (4–19)	0.711
Maximum serum creatinine^b^ (mg/dL) between enrollment and time of first sample collection after onset of AKI Stage 2 or 3	1.3 (0.9–1.8)	1.2 (0.8–1.6)	2.4 (1.4–3.2)	**< 0.001**
Time between enrollment and first urinary CCL14 sample collection (h)	29 (15–60)	33 (15–60)	21 (0–34)	0.057

^a^Fluid balance was defined as the difference in total fluid intake minus output from the day prior to through the day of study enrollment

^b^Specifically, this is the greatest (maximum) of all serum creatinine concentrations (mg/dL) collected as standard of care between the time of study enrollment and the time of the first urine sample collection for CCL14 testing after the onset of AKI Stage 2 or 3. This is expressed as a median (interquartile range)

^c^*P* values for age, baseline serum creatinine, non-renal APACHE III score, time from ICU admission to enrollment, serum creatinine at time of first sample collection after onset of AKI Stage 2 or 3, and fluid balance were computed by the Mann–Whitney U test. All others were computed by Fisher’s Exact Test. *P *values less than 0.05 are in bold

### CCL14 and persistent severe AKI

**Fig. 2:**
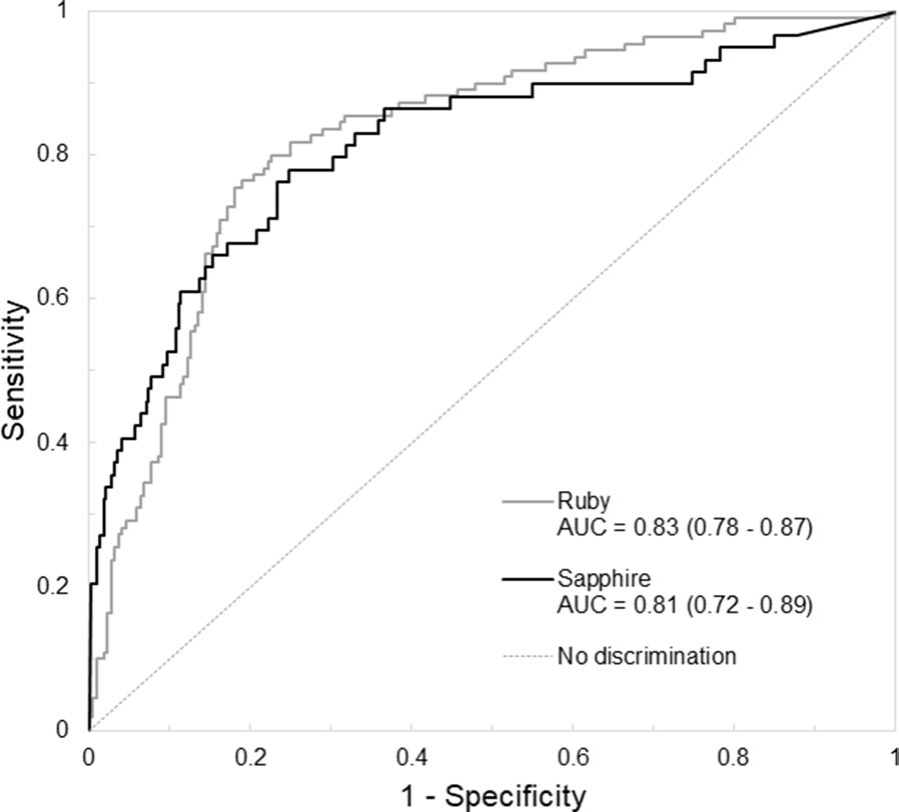
Receiver operating characteristic curves for prediction of persistent severe AKI with urinary CCL14 in the RUBY (solid gray) [6] and SAPPHIRE (solid black) [9] studies. Dashed gray reference line shows no discrimination between persistent severe AKI and no persistent severe AKI

**Fig. 3:**
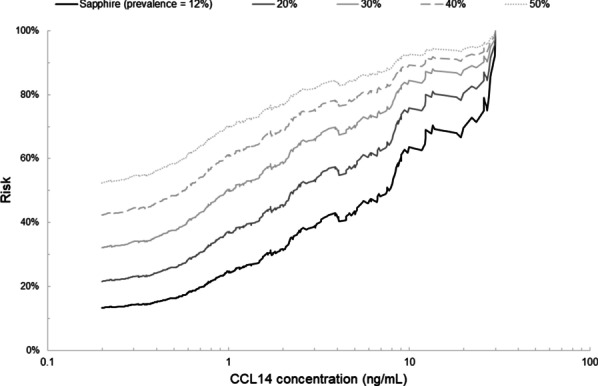
Risk for persistent severe AKI versus CCL14 concentration observed in SAPPHIRE (solid black) and simulated at different levels of persistent severe AKI: 20% (solid dark gray), 30% (solid gray), 40% (dashed gray), and 50% (dotted gray)

Urinary CCL14 showed good prediction for the occurrence of persistent severe AKI with an AUC 0.81 (95% CI, 0.72–0.89) (Fig. 2). The AUC was not significantly different from the AUC in the RUBY study [6]. The risk for persistent severe AKI increased with greater values of urinary CCL14 (Fig. 3). This increased risk was consistent across simulated curves that modified the baseline prevalence of AKI in the SAPPHIRE population. Urinary CCL14 improved the predictive ability of clinical variables evaluated with GEE. The addition of urinary CCL14 to a baseline clinical model that included serum creatinine and non-renal APACHE III score for the development of persistent severe AKI provides incremental predictive information beyond clinical variables alone (Table 2). This was similarly shown in sensitivity analysis adjusting instead for trajectory in serum creatinine (Additional file 1: S3). In a further sensitivity analysis, we explored the effect of oliguria on the performance of urinary CCL14 to predict persistent severe AKI and found no evidence of significant interaction (Additional file 1: S4).

**Table 2:** Integrated discrimination improvement (IDI), category-free net reclassification improvement (cfNCI), and AUC difference with the addition of CCL14 to a reference clinical model with baseline serum creatinine and non-renal APACHE III score

	Value	95% CI	*P *value
IDI	0.065	0.005–0.172	< 0.001
IDI: event	0.048	0.003–0.141	< 0.001
IDI: non-event	0.017	0.002–0.035	< 0.001
cfNRI	0.864	0.389–1.276	< 0.001
cfNRI: event	0.322	0.051–0.633	0.004
cfNRI: non-event	0.542	0.272–0.713	< 0.001
AUC ref model	0.768	0.662–0.872	< 0.001
AUC new model	0.834	0.738–0.918	< 0.001
AUC difference	0.065	0.007–0.143	< 0.001

Model	Variable	OR	95% CI	*P* value
Reference	Non-renal APACHE	1.85	1.25–2.73	0.002
	Baseline serum creatinine	2.26	1.42–3.62	0.001
New	Non-renal APACHE	1.63	1.12–2.39	0.012
	Baseline serum creatinine	2.09	1.24–3.50	0.005
	CCL14	1.63	1.21–2.19	0.001

Event is persistent severe AKI. Odds ratio (OR), 95% confidence interval (CI), and *P* value for each variable in the models are shown in the lower table

### CCL14 and mortality and RRT

**Fig. 4:**
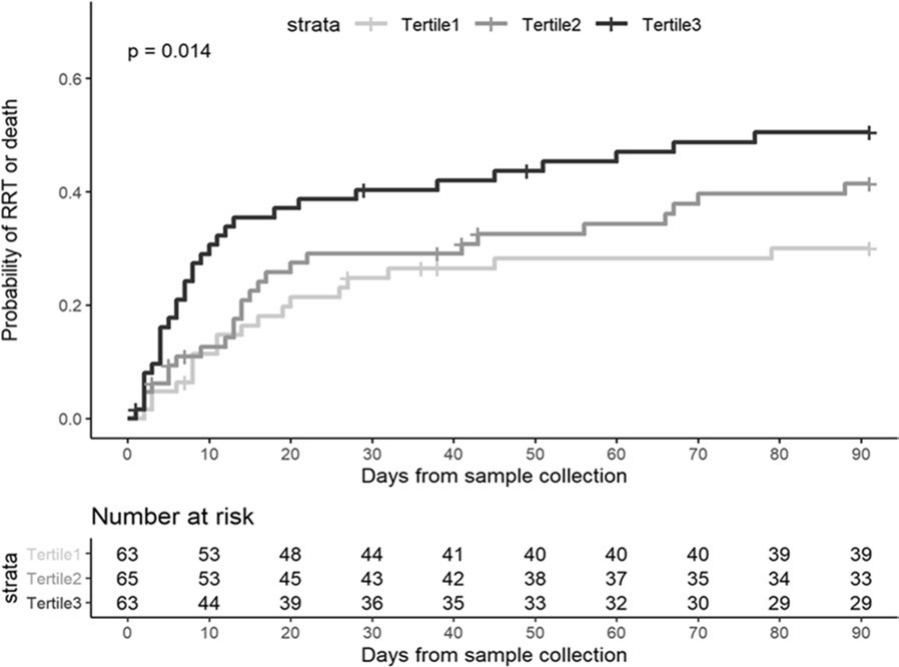
Cumulative incidence probability of RRT or death within 90 days of the first sample collection after onset of stage 2 or 3 AKI stratified by CCL14 concentration tertiles. Tertile 1 (light gray), Tertile 2 (dark gray), and Tertile 3 (black) correspond to concentrations at or below the 33rd percentile, between the 33rd and 67th percentile, and above the 67th percentile of all CCL14 concentrations. *P *value = 0.014 for the log rank test

RRT use, death and a composite of RRT and/or death occurred in 29 (15%), 63 (32%) and 74 (38%) patients within 90 days, respectively (Additional file 1: S5). The probability of RRT and/or death within 90 days increased across tertiles of urinary CCL14 concentration (Fig. 4). This was shown in the unadjusted models for urinary CCL14 both as a continuous variable and by tertiles; as well as following adjustment for baseline serum creatinine and non-renal APACHE III score when comparing CCL14 values from tertile 3 to tertile 1 (Table 3).

**Table 3:** Hazard ratio for (A) continuous CCL14 and (B) CCL14 tertiles, unadjusted and adjusted for baseline serum creatinine and non-renal APACHE III score in a Cox regression model with CCL14 as a time-dependent covariate

Model	HR (95% CI)	*P* value
*A. Urinary CCL14 concentration as a continuous variable in model*		
Unadjusted (CCL14 only)	1.67 (1.36–2.06)	< 0.001
Adjusted for baseline serum creatinine	1.55 (1.24–1.93)	< 0.001
Adjusted for baseline non-renal APACHE	1.51 (1.22–1.88)	< 0.001
Adjusted for baseline serum creatinine and non-renal APACHE	1.41 (1.13–1.75)	0.003

Model	Tertile 2 versus 1		Tertile 3 versus 1	
	HR (95% CI)	*P* value	HR (95% CI)	*P *value
*B. Urinary CCL14 concentration divided into tertiles and used as a categorical variable in model*				
Unadjusted (CCL14 only)	1.14 (0.61–2.13)	0.671	2.56 (1.46–4.50)	0.001
Adjusted for baseline serum creatinine	1.09 (0.58–2.02)	0.795	2.16 (1.20–3.88)	0.010
Adjusted for baseline non-renal APACHE	1.15 (0.62–2.14)	0.662	2.10 (1.18–3.75)	0.012
Adjusted for baseline serum creatinine and non-renal APACHE	1.15 (0.62–2.15)	0.651	1.83 (1.02–3.29)	0.043

Model is for time to RRT and/or death within 90 days

## Discussion

We performed a secondary analysis using the multi-national prospective observational SAPPHIRE study to externally validate the ability of the novel biomarker urinary CCL14 to predict persistent severe AKI, defined as at least 72 h of stage 3 AKI, receipt of RRT or death, among a heterogeneous cohort of critically ill patients with stage 2–3 AKI. We found that urinary CCL14 had good predictive performance for the development of persistent severe AKI (AUC 0.81), was not modified by oliguria, and provided incremental value beyond commonly available clinical variables. We also found urinary CCL14 correlated with death and RRT use in unadjusted and in adjusted analysis when comparing CCL14 from the highest and lowest tertiles. Our analysis largely replicated the performance for urinary CCL14 with the findings of the RUBY study (AUC 0.83) and now provides added knowledge to extend our confidence in the validity and potential role urinary CCL14 can have in clinical practice [6]. Furthermore, we have extended the findings of the RUBY study by validating the clinical assay using a commercially available analyzer and we have further investigated the relationship between CCL14 and persistent severe AKI by simulating different baseline prevalence rates. We submit that our findings provide a further foundation for evaluation of the application and use of urinary CCL14 in clinical practice.

CCL14 is hypothesized to be an important mediator of kidney tissue damage and may represent a novel indicator of the risk for maladaptive repair and kidney non-recovery [6]. Prior work has shown CCL14 is one of a series of important “kidney risk inflammatory signature [KRIS]” inflammatory proteins circulating in patients with DM which may mediate the risk of progressing to end-stage kidney disease (ESKD) [7]. The mechanisms by which CCL14 mediates persistent severe AKI are not yet completely understood. In AKI, CCL14 may be released from the injured tubular epithelial cells through the activation of inflammatory mediators (e.g., via stimulation of TNF-a receptor activity). However, we submit that further translational work is needed to better understand the role of CCL14 in potentially disrupting intrinsic kidney repair mechanisms following an episode of AKI, how this may contribute to persistent severe AKI and downstream risk of new or worsening CKD and ESKD.

Our external validation of urinary CCL14 to predict the occurrence of persistent severe AKI has several noteworthy implications. From a research standpoint, urinary CCL14 may represent an innovative mechanistic surrogate endpoint used to evaluate novel therapeutic targets for the prevention of persistent severe AKI and its sequelae [5]. For example, CCL14 may be a critical chemokine for monocyte/macrophage recruitment and activation and may mediate kidney injury and accelerate kidney fibrosis [12, 13]. In this context, CCL14 may also be a target for modulation or inhibition in clinical trials. In addition, urinary CCL14 can further be used to discriminate eligibility thresholds to predictively enrich the target population in clinical trials that aim to evaluate therapeutic strategies in patients at high risk for experiencing persistent severe AKI and its sequelae [14].

From a clinical perspective, urinary CCL14 can provide incremental and valuable information for clinicians that can translate into improved quality of care at the bedside. For example, a positive urinary CCL14 test can better advise clinician’s expectations about the clinical course in a given patient and directly inform care processes, including triaging to a suitable level of monitoring (e.g., ICU or HDU; serial serum creatinine and urine output), augmented surveillance for and avoidance of complications (e.g., withholding potassium supplementation, modifications to enteric nutrition, fluid overload, etc.), and dose-modification of or overt avoidance of selected medications with potential toxic or cumulative adverse effects. Alternatively, a negative urinary CCL14 could provide reassurance about de-intensification of monitoring.

Similarly, knowledge of greater risk for persistent severe AKI should provoke nephrology consultation and planning for the potential receipt of RRT for those with further deterioration. Conversely, a negative urinary CCL14 could reinforce a “watch and wait strategy” and prompt reconsideration of early start to RRT unless absolute indications are present that are medically refractory and unavoidable [8].

Finally, a positive CCL14 should prompt consideration for the long-term implications of incomplete kidney recovery and risk of incident CKD, progression to ESKD and other complications associated with AKI [15–19]. Our data now reinforce existing recommendations in clinical practice guidelines that survivors of AKI have a routine assessment of kidney function at three months and as needed thereafter, whereby such risk and complications can be identified and potentially modified [20].

Our study has strengths. This study leveraged a high-fidelity international prospective observational cohort for external validation. The study integrated current consensus recommendations for the diagnosis and staging of AKI [20]. The main study exposure, urinary CCL14, was blinded to clinicians during the study and was evaluated from high-quality stored samples. However, our study also has limitations. The number of patients experiencing the primary outcome in our validation study was small (14%); and as such, the study is certain to have limited statistical power. This is particularly true for analyses of secondary endpoints, which should be viewed as exploratory and hypothesis generating. Further, we recognize that the SAPPHIRE study was performed several years ago and acknowledge that temporal changes in clinical practice (e.g., indications and timing for RRT) may have evolved in response to new data [8, 21–23]. Additionally, the study did not further classify AKI events into sub-phenotypes which also may have important prognostic implications and consideration for case-mix selection for future clinical trials.

## Conclusions

Our study externally validated urinary CCL14 to predict persistent severe AKI. The findings of an elevated urinary CCL14 in critically ill patients with AKI have relevance for clinical care and represent a mechanism of predictive enrichment for clinical trials.

## Supplementary Information

**Additional file 1.** Supplementary data presentation and analysis to support the main analysis.

## Data Availability

Requests for access to the SAPPHIRE dataset can be made to Astute Medical.

## Abbreviations

AKI: Acute kidney injury
APACHE: Acute Physiology and Chronic Health Evaluation
AUC: Area under receiver operating characteristic curve
CCL14: C–C motif chemokine ligand 14
COPD: Chronic obstructive pulmonary disease
cfNRI: Category-free net reclassification improvement
CKD: Chronic kidney disease
DM: Diabetes mellitus
ESKD: End-stage kidney disease
GFR: Glomerular filtration rate
HR: Hazard ratio
ICU: Intensive care unit
IDI: Integrated discrimination improvement
KDIGO: Kidney Disease: Improving Global Outcomes
RCT: Randomized controlled trial
RRT: Renal replacement therapy
