# Supplementary material for: External validation of urinary C–C motif chemokine ligand 14 (CCL14) for prediction of persistent acute kidney injury
Source: Crit Care. 2021 May 31;25:185. doi: 10.1186/s13054-021-03618-1 (PMC8166095; doi:10.1186/s13054-021-03618-1)
Supplement: Supplementary file 1 — Additional file 1. Supplementary data presentation and analysis to support the main analysis. [file 13054_2021_3618_MOESM1_ESM.docx]

**Additional Files**

**Additional File 1.** Comparison of mean (A) and median serum creatinine concentrations (B) between persistent (solid black) and not persistent (dashed black) patients from enrollment to 7 days after. Dotted gray line shows the percentage of patients with data available at each hour.

(A)

(B)

**Additional File 2.** Comparison of mean (A) and median urine output (B) between persistent (solid black) and not persistent (dashed black) patients from enrollment to 7 days after. Each data point represents a 6-hour moving average of the statistic. Dotted gray line shows the percentage of patients with data available at each hour.

(A)

(B)

**Additional File 3.** Integrated discrimination improvement (IDI), category-free net reclassification improvement (cfNCI), and AUC difference with the addition of CCL14 to a reference clinical model using serum creatinine trajectory and non-renal APACHE III score. Event is persistent severe AKI. Odds ratio (OR), 95% confidence interval (CI), and p-value for each variable in the models are shown in the lower table.

|  | **Value** | **95% CI** | **p-value** |
| --- | --- | --- | --- |
| IDI | 0.052 | 0.010 - 0.239 | <0.001 |
| IDI: event | 0.038 | 0.004 - 0.204 | <0.001 |
| IDI: non-event | 0.014 | 0.002 - 0.043 | <0.001 |
| cfNRI | 1.157 | 0.502 - 1.346 | <0.001 |
| cfNRI: event | 0.661 | 0.115 - 0.778 | <0.001 |
| cfNRI: non-event | 0.496 | 0.201 - 0.700 | <0.001 |
| AUC ref model | 0.694 | 0.605 - 0.813 | <0.001 |
| AUC new model | 0.802 | 0.718 - 0.919 | <0.001 |
| AUC difference | 0.109 | 0.020 - 0.236 | <0.001 |

| **Model** | **Variable** | **OR** | **95% CI** | **p-value** |
| --- | --- | --- | --- | --- |
| Reference | Non-renal APACHE | 1.86 | 1.27 - 2.75 | 0.002 |
|  | Serum creatinine trajectory | 1.07 | 0.96 - 1.20 | 0.217 |
| New | Non-renal APACHE | 1.64 | 1.12 - 2.40 | 0.011 |
|  | Serum creatinine trajectory | 1.11 | 0.95 - 1.29 | 0.183 |
|  | CCL14 | 1.38 | 1.13 - 1.69 | 0.002 |

**Additional File 4.** Interaction between oliguria and CCL14 using GEE regression with persistent AKI as the dependent variable. Oliguria is a categorical variable with 2 strata: those patients with KDIGO Stage 1 – 3 by urine output criteria vs. those with no AKI by KDIGO urine output criteria.

| **Variable** | **OR** | **95% CI** | **p-value** |
| --- | --- | --- | --- |
| CCL14 | 1.90 | 1.40 - 2.59 | <0.001 |
| Oliguria | 0.82 | 0.61 - 1.09 | 0.170 |
| Oliguria x CCL14 | 1.25 | 0.87 - 1.81 | 0.227 |

**Additional File 5.** Number of subjects who had RRT or died without RRT within 90 days by CCL14 concentration tertiles.

|  | **Tertile 1** | **Tertile 2** | **Tertile 3** | **Total** |
| --- | --- | --- | --- | --- |
| **RRT** | 6 (33%) | 5 (20%) | 18 (58%) | 29 (39%) |
| **non-RRT Death** | 12 (67%) | 20 (80%) | 13 (42%) | 45 (61%) |
| **RRT or death** | 18 (100%) | 25 (100%) | 31 (100%) | 74 (100%) |
